# Autophagy: An Intracellular Degradation Pathway Regulating Plant Survival and Stress Response

**DOI:** 10.3389/fpls.2020.00164

**Published:** 2020-02-28

**Authors:** Tong Su, Xuezhi Li, Mingyue Yang, Qun Shao, Yanxiu Zhao, Changle Ma, Pingping Wang

**Affiliations:** Shandong Provincial Key Laboratory of Plant Stress, College of Life Sciences, Shandong Normal University, Jinan, China

**Keywords:** autophagy, selective autophagy, receptor, stress, plant

## Abstract

Autophagy is an intracellular process that facilitates the bulk degradation of cytoplasmic materials by the vacuole or lysosome in eukaryotes. This conserved process is achieved through the coordination of different *autophagy-related* genes (*ATGs*). Autophagy is essential for recycling cytoplasmic material and eliminating damaged or dysfunctional cell constituents, such as proteins, aggregates or even entire organelles. Plant autophagy is necessary for maintaining cellular homeostasis under normal conditions and is upregulated during abiotic and biotic stress to prolong cell life. In this review, we present recent advances on our understanding of the molecular mechanisms of autophagy in plants and how autophagy contributes to plant development and plants’ adaptation to the environment.

## Introduction

Autophagy, which means self-eating, is found universally in all eukaryotic cells. It is an important process for degrading proteins and organelles, *via* the vacuole in yeast and plants or through the lysosome in animals, to facilitate intracellular recycling (Marshall and Vierstra, 2018). In plants, autophagy is continuously maintained at a basal level during growth and development to ensure homeostasis, but is upregulated under environmental stresses to aid plant survival (Wang et al., 2018a). Three major types of autophagy have been described in plants: microautophagy, macroautophagy, and mega-autophagy. Among these, macroautophagy has been the best studied (van Doorn and Papini, 2013).

During microautophagy, cytosolic proteins or entire organelles are congregated together near the vacuole and become encapsulated by an invagination or protrusion of the vacuolar membrane. This forms an intravacuolar vesicle called an autophagic body, which is released to the vacuolar lumen by membrane scission and is degraded (van Doorn and Papini, 2013; Marshall and Vierstra, 2018). In contrast, in macroautophagy, a cup-shaped double-membrane structure, named the phagophore, is formed at the phagophore assembly site (PAS). Some studies show that the phagophore arises from endoplasmic reticulum (ER), mitochondria and plasma membrane or from ER-mitochondria contact sites (Hamasaki et al., 2013; Le Bars et al., 2014; Zhuang et al., 2017). The phagophore elongates and encircles the cytosolic substances to form double membrane-bound autophagosomes that subsequently fuse with the vacuole to release the internal vesicle for degradation (Liu and Bassham, 2012). The third route of autophagy, which is only found in plants, is mega-autophagy and occurs concomitantly with programmed cell death (PCD). Large amounts of hydrolases are released into the cytoplasm from the vacuole, resulting in large-scale degradation of cellular components including cytoplasm, all organelles, the plasma membrane and part of the cell wall (Hara-Nishimura and Hatsugai, 2011; van Doorn and Papini, 2013). Unlike both microautophagy and macroautophagy that recycle macromolecular constituents back to the cytosol from the vacuole, mega-autophagy is an extreme form of massive degradation resulting in cell death.

In addition to the conserved microautophagy and macroautophagy pathways, yeast also have a cytoplasm-to-vacuole targeting (Cvt) pathway that is biosynthetic and occurs constitutively to deliver precursors of resident hydrolases to the vacuole. The Cvt pathway operates under a similar mechanism to macroautophagy (Klionsky and Emr, 2000). In mammals, chaperone-mediated autophagy is the third pathway used for transferring substrate proteins directly to the lysosome without the use of separate vesicles (Dice, 2007). However, no evidence has been shown that the Cvt pathway nor chaperone-mediated autophagy exist in plants.

Macroautophagy is characterized by the presence of autophagosomes with a double-membrane envelope that form at the PAS and will eventually fuse with the vacuole. A series of *autophagy-related* genes (*ATGs*) are responsible for the initiation and formation of autophagosomes. Although autophagy has long been regarded as a process of nonselective degradation of cellular structures (general autophagy), plentiful evidence indicates that autophagy is also a highly selective mechanism to target a wide range of superfluous or damaged components as part of cellular quality control and stress responses (selective autophagy) (Yoshimoto and Ohsumi, 2018). Many ATG proteins involved in general autophagy are conserved in yeast, plants, and animals, indicating that eukaryotes may share similar autophagy mechanisms. In contrast, the selective autophagy pathways rely on a variety of selective autophagy receptors. Although the selective autophagy receptors are not conserved, their modes of action and regulatory mechanisms are similar across eukaryotes (Farré and Subramani, 2016). Various autophagy pathways have specific roles during plant development and stress response. In this review, we summarize recent advances in understanding the molecular mechanisms underlying autophagy in plants and the important roles that autophagy plays in plant development and stress responses.

## Autophagy in Plants

### The Molecular Mechanism of Autophagy

Although autophagosomes were first observed in mammalian cells in the 1950s (de Duve et al., 1955), the molecular mechanisms of autophagy have been primarily revealed from research in yeast, then extended to animal and plant (Tsukada and Ohsumi, 1993; Ohsumi, 2001; Marshall and Vierstra, 2018). These great progresses expand our understanding to higher eukaryotes in recent decades.

**Figure 1:**
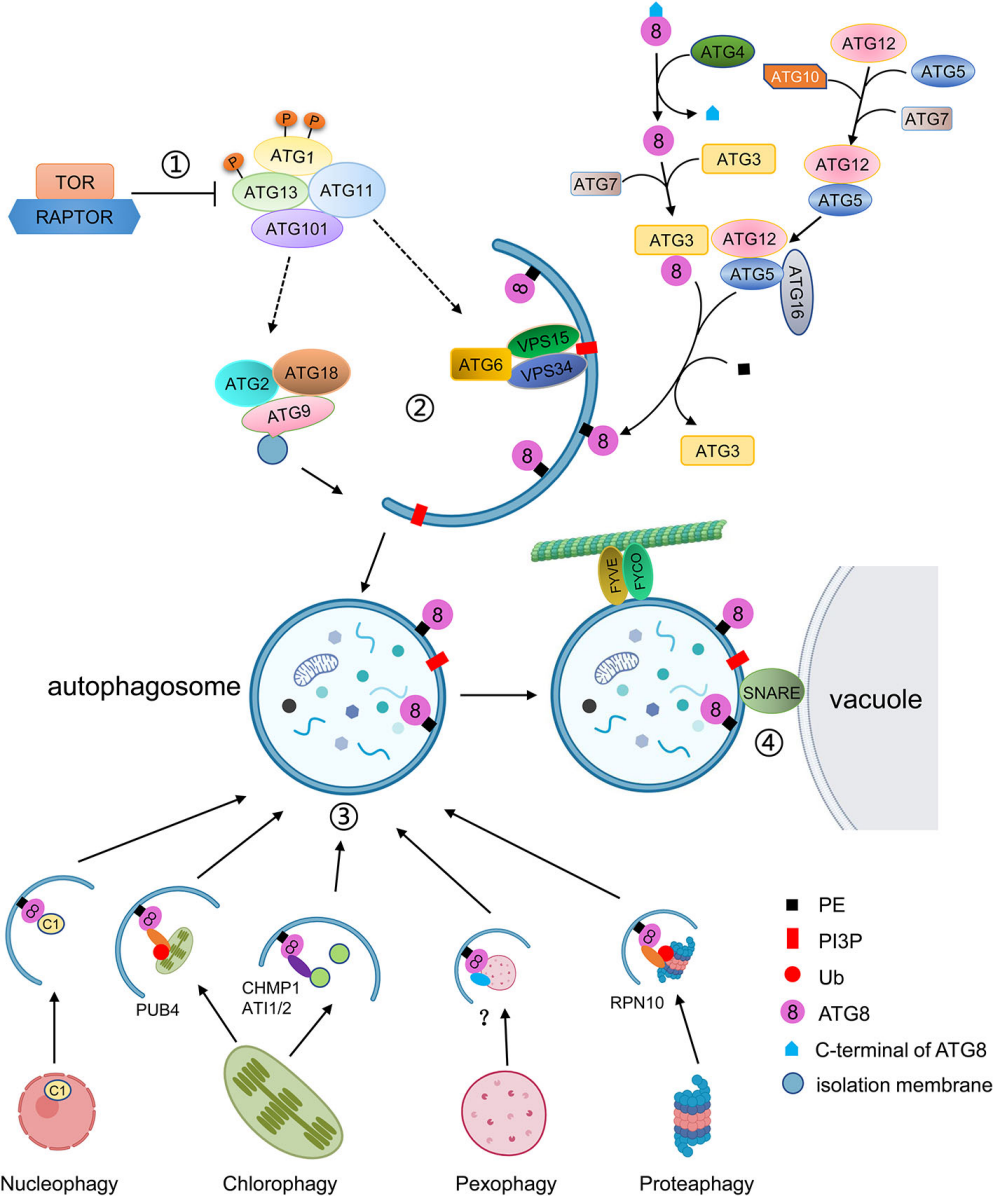
Schematic diagram of the autophagy process. ① Autophagy is regulated by TOR kinase. Inhibition of TOR results in activation of constitutive autophagy, while overexpression of TOR blocks autophagy. Autophagy is initiated by the association of ATG13 and ATG1, together with ATG11 and ATG101, into an active complex. ② Autophagosome formation includes membrane delivery, vesicle nucleation and phagophore expansion and closure. ATG9, with ATG2 and ATG18, is involved in the delivery of lipids to the expanding phagophore. The VPS34 lipid kinase complex generates PI3P decoration, which is accompanied by the conjugation of ATG8 to PE. ATG8 firstly is matured by cleavage of its C-terminal by ATG4, and then is conjugated to PE by E2-like ATG3 and the E3-like ATG12-ATG5-ATG16 complex. ATG8-PE localizes to the autophagosomal membrane for phagophore expansion. ③ Selective autophagy is mediated by interaction of ATG8 with specific autophagic receptors through the AIM domain. Different cell components can be recognized by specific autophagic receptors. For instance, the C1 protein is translocated from the nucleus to the cytoplasm and binds to ATG8 for degradation. The whole chloroplast is ubiquitulated by PUB4 and binds to ATG8. The fragments of chloroplasts are recognized by CHMO1 or ATI1/2 for degradation by RCBs or ATI1/2 decorated plastid bodies. In plants, peroxisomes to be degraded are probably recognized by PEX6 or PEX10, and the ubiquitylated proteasome is recognized by RPN10. ④ The mature autophagosome is transported to the vacuole with the help of FYVE and FYCO proteins and fused with the vacuole to be degraded.

Macroautophagy, often simply called autophagy, has been well characterized (**Figure 1**). The autophagy process can be divided into distinct stages: induction, cargo recognition, phagophore formation, phagophore expansion and closure, and autophagosome fusion and breakdown (Masclaux-Daubresse et al., 2017). More than 40 *ATG* genes have been characterized in yeast. Homologs of many *ATGs* have been characterized in animals and plants (Tang and Bassham, 2018). At present, about 40 *ATGs* have been identified in *Arabidopsis thaliana*, most of which are homologous with yeast *ATGs* (Chung, 2019). The ATG proteins can be divided into four core functional groups: 1) the ATG1/ATG13 kinase complex that initiates autophagosome formation in response to nutrient limitation; 2) the autophagy-specific class III phosphatidylinositol (PI) 3-kinase complex; 3) the ATG9 complex which promotes phagophore expansion; and 4) the ATG8/ATG12 ubiquitin-like conjugation systems which act during phagophore expansion and maturation (Kim et al., 2012; Wang et al., 2018a).

Autophagy is initiated by the down-regulation of the TOR (Target of Rapamycin) complex as a consequence of various stimuli, such as developmental and nutritional signals (Marshall and Vierstra, 2018). TOR is an essential serine/threonine kinase belonging to the phosphatidylinositol kinase-related kinase family and negatively regulates autophagy (Díaz-Troya et al., 2008). In yeast, TOR1/2, KOG1 (Kontroller of growth 1), LST8 (Lethal with Sec13 protein 8), and TCO89 (89-kDa subunit of Tor complex 1) form TOR complex 1 (TORC1) to inhibit autophagy (Díaz-Troya et al., 2008; Cebollero and Reggiori, 2009; He and Klionsky, 2009). It has been reported that the TORC1 equivalent also exists in plants (Robaglia et al., 2012). In *Arabidopsis*, TOR is encoded by a single gene, which is essential for embryo development (Menand et al., 2002). The *Arabidopsis* RAPTOR family is homologous to yeast KOG1 and functions as the target recognition cofactor of TOR. The RAPTOR family consists of RAPTOR1/Raptor1B and RAPTOR2/Raptor1A. Disruption of RAPTOR1/Raptor1B causes repression of embryo development and post-embryonic plant growth, whereas disruption of RAPTOR2/Raptor1A does not affect plant development and growth (Anderson et al., 2005; Deprost et al., 2005). In *Arabidopsis,* two putative homologs of LST8 have been identified; however, no TCO89 homologs have yet been found (Díaz-Troya et al., 2008; Moreau et al., 2012). As regulatory associated proteins, the LST8 homologs may be involved in plant autophagy by regulating TOR activity, but this needs to be confirmed. In plants, active TOR inhibits autophagy under normal conditions, whereas inactive TOR promotes autophagy under nutrient starvation or during treatment with chemical inhibitors such as rapamycin or AZD8055 (Ren et al., 2012; Li et al., 2015b).

*Arabidopsis* SnRK1 (SNF1/AMPK-related kinase 1), the homolog of SNF1/AMPK, which can inhibit TOR activity in yeast and mammals, is a heterotrimeric complex consisting of a catalytic α subunit and regulatory β and γ subunits (Smeekens et al., 2010). KIN10 is one of the three isoforms of the SnRK1 catalytic subunit involved in autophagy. Autophagy is blocked in mutants lacking *KIN10* under nutrient deficiency and salt, oxidative, and ER stress conditions, whereas overexpression of *KIN10* induces autophagy under abiotic stresses (Chen et al., 2017; Soto-Burgos and Bassham, 2017). Under nutrient deprivation, KIN10 promotes the phosphorylation of ATG1 and the TOR complex subunit RAPTOR to inhibit TOR activity and initiate autophagy (Nukarinen et al., 2016; Chen et al., 2017).

In *Arabidopsis*, under normal conditions, TOR is active and hyperphosphorylates ATG13. Highly phosphorylated ATG13 has a low binding capacity for ATG1, resulting in low activity of ATG1 and maintenance of basal levels of autophagy. In *Arabidopsis*, ATG1 and ATG13, which along with other two accessory subunits ATG11 and ATG101, are assembled into an active complex to initiate autophagy (Suttangkakul et al., 2011; Li and Vierstra, 2014). However, whether the assembly of the ATG1-ATG13 complex is regulated by nutrient availability is still under debate.

In *Arabidopsis*, the activation of the ATG1-ATG13 complex stimulates the downstream steps of autophagy including membrane delivery, vesicle nucleation, and phagophore expansion and closure (Li and Vierstra, 2012; Marshall and Vierstra, 2018). ATG9, along with ATG2 and ATG18, is involved in the formation of the isolation membrane at the PAS and the delivery of lipids to the expanding phagophore (Zhuang et al., 2017). In yeast and mammals, *atg9* mutants fail to form autophagosomes, but in *Arabidopsis*, the deletion of *ATG9* results in expanding autophagosome-related tubules connected to the ER (Orsi et al., 2012; Yamamoto et al., 2012; Zhuang et al., 2017). Also, the sequence of AtATG9 shows limited homology with those of yeast and mammals, suggesting that AtATG9 may function in a plant-specific manner during autophagosome formation (Zhuang et al., 2018).

In yeast, the Atg2–Atg18 complex is localized to the PAS in a phosphatidylinositol-3-phosphate (PI3P)-dependent manner with Atg2 associating with the ER to form the isolation membrane (Kotani et al., 2018). In yeast, PI3P, a small membrane lipid, is generated by the class III PI3K (phosphatidylinositol-3-kinase) complex I, consisting of Vps34, Vps15, Vps30/Atg6, and Atg14 (Kihara et al., 2001). During autophagy, PI3K complex I localizes to the PAS to recruit downstream proteins through its product, PI3P. Loss of *Arabidopsis* VPS34, ATG6/VPS30, and VPS15, cause lethality due to defects in pollen germination and development (Fujiki et al., 2007; Lee et al., 2008; Xu et al., 2011). Heterozygous *Arabidopsis vps34* and *vps15* mutants display abnormal vacuole morphology (Lee et al., 2008). In tobacco cell culture, PI3K inhibitors such as wortmannin, LY294002, and 3-methyladenine (3-MA) impair the formation of autophagosomes and block the accumulation of autolysosomes, indicating that PI3K plays a crucial role in autophagy (Takatsuka et al., 2004).

Meanwhile, a ubiquitin-like conjugation pathway is required for the insertion of ATG8-phosphatidylethanolamine (PE) into the membrane of the expanding phagophore (Marshall and Vierstra, 2018). In yeast, Atg8 is initially synthesized as a long, inactive precursor, which is subsequently cleaved by the cysteine protease Atg4 to expose a glycine residue on the C-terminus (Ohsumi, 2001). The mature ATG8 can be activated through binding of ATP-dependent E1 (ubiquitin-activating enzyme)-like ATG7 to its newly exposed glycine. ATG8 is subsequently donated to the E2 (ubiquitin-conjugating enzyme)-like ATG3 and finally conjugated to PE by the E3 (ubiquitin ligase)-like ATG12-ATG5-ATG16 complex. This complex is formed by another ubiquitin-fold protein, ATG12, that is conjugated to ATG5 through the sequential actions of ATG7 and the ATG12-specific E2 conjugating enzyme ATG10. ATG12-ATG5 then connects to the dimeric ATG16 protein to tether to the phagophore, allowing for the lipidation of ATG8 with PE (Hanada et al., 2007; Chung et al., 2010).

The ATG8 ubiquitin-like conjugation pathway was also been reported to exist in *Arabidopsis*. However, unlike in yeast, *Arabidopsis* has nine homologs of *ATG8* (*ATG8a-ATG8i),* two homologs of *ATG4* (*ATG4a* and *ATG4b*), and two homologs of *ATG12* (*ATG12a* and *ATG12b*) (Doelling et al., 2002; Hanaoka et al., 2002). The *Arabidopsis ATG8* genes have different expression patterns, indicating that they may have distinct functions (Sláviková et al., 2005). In *Arabidopsis*, the ATG4s can cleave the C-terminus of ATG8, as its homolog does in yeast. Moreover, the *atg4a atg4b* double mutant displays autophagy defects, evidenced by early senescence and decreased silique production, indicating that ATG4s are essential for plant development (Yoshimoto et al., 2004). In *Arabidopsis*, the *atg12a atg12b* double mutant shows premature senescence, sensitivity to nutrient starvation, and lack of autophagic bodies, whereas the single *atg12a* or *atg12b* mutants do not. The accumulation of the ATG12-ATG5 conjugate was reduced in either *atg12a* or *atg12b* single mutants, and ATG8-PEs were not detected in *atg12a atg12b*, indicating that the ATG12-ATG5 conjugate is essential for ATG8-PE conjugations (Chung et al., 2010). Mutation of *ATG5*, *ATG7* or *ATG10* results in plants which are hypersensitive to nitrogen and carbon starvation (Chung et al., 2010). Like *atg12*, the *atg10-1* and *atg5-1* mutants fail to form autophagic bodies in the vacuole (Phillips et al., 2008).

The ATG8-PE conjugates, which are located in both the inner and outer autophagosome membranes, aid phagophore expansion and vesicle closure to form autophagosomes and recognize autophagic cargoes through ATG8-interacting proteins (Johansen and Lamark, 2011; Liu and Bassham, 2012). In *Arabidopsis*, a membrane associated protein SH3P2 (SH3 domain-containing protein 2) translocates to the PAS during autophagy (Zhuang et al., 2013). SH3P2 not only interacts with ATG8 but also binds to PI3P and associates with the PI3K complex to facilitate membrane elongation and autophagosome closure (Zhuang et al., 2013).

In mammals, when the autophagosome is sealed, FYCO1 (FYVE and coiled-coil domain containing 1) binds to LC3/ATG8 and PI3P on the outer autophagosome membrane to promote autophagosome movements through microtubule plus end-directed transport (Pankiv et al., 2010). In *Arabidopsis*, co-sedimentation and co-localization assays showed that ATG8 can bind to microtubules *in vivo*, suggesting microtubules may be involved in relocation of autophagosomes to the vacuole (Ketelaar et al., 2004).

Correct targeting of autophagosomes to the vacuole requires SNAREs (soluble NSF attachment protein receptors) (Surpin et al., 2003). In *Arabidopsis*, under nutrient starvation conditions, loss of VTI12, a VTI1-type v-SNARE (vesicle-SNARE) located on the target membrane, results in failure of the autophagosomes to enter the vacuole, indicating that VTI12 plays an important role in autophagosome fusion with the tonoplast (Surpin et al., 2003).

Autophagosome fusion and degradation require the ESCRT (Endosomal Sorting Complex Required for Transport) machinery. Recently, some of the components involved in this process have been identified. *Arabidopsis* AMSH3 (associated molecule with the SH3 domain of STAM3), a deubiquitinating enzyme, interacts with the ESCRT-III subunit VPS2.1 (vacuolar protein sorting 2.1) and is essential for autophagosome trafficking to the vacuole (Katsiarimpa et al., 2013). A plant-specific ESCRT component, FREE1 (FYVE domain protein required for endosomal sorting 1), was reported to directly interact with the autophagy regulator SH3P2 and to play a role in autophagosome-vacuole fusion in *Arabidopsis* (Zhuang et al., 2013; Gao et al., 2015). *Arabidopsis* CFS1 (Cell death related endosomal FYVE/SYLF protein 1), a FYVE and SYLF domain-containing protein unique to plants that co-localizes and interacts with PI3P and the ESCRT-I component ELCH, is enriched in the autophagosome membrane and participates in autophagosome turnover (Sutipatanasomboon et al., 2017).

After the autophagosome and vacuole are fused, the internal vesicle, called the autophagic body, is released into the vacuole and degraded by a series of resident hydrolases (Marshall and Vierstra, 2018). ATG8-PE on the inner autophagosome membrane is also degraded in the vacuole, while the ATG8-PE attached to the outer autophagosome membrane is cleaved by ATG4 to release ATG8 from PE for recycling (Yoshimoto et al., 2004).

### Types of Selective Autophagy

Autophagy occurs during all developmental stages of plants. It has become increasingly clear that selective autophagy also exists to specifically remove certain cellular components. The specificity of autophagy is mediated by interactions between ATG8 and specific autophagic receptors containing an ATG8-interacting motif (AIM) (Marshall et al., 2015; Farré and Subramani, 2016; Maqbool et al., 2016). Autophagic receptors mediate selective autophagy of specific cellular components, such as various organelles, protein aggregates, and even invading pathogens (Marshall and Vierstra, 2018). Selective autophagy endows plants with the ability to specifically regulate cell activity according to their physiological state and external environment.

#### Proteaphagy

In plants, the quality control machinery, in which the ubiquitin-proteasome system (UPS) plays an important role, is utilized to degrade defective proteins and maintain the integrity of the cellular proteome (Balchin et al., 2016). Selective autophagy also plays an important role in the clearance of damaged protein complexes, such as proteasomes (Yoon and Chung, 2019). Proteaphagy was first described in plants and is activated by two pathways: one is induced by nitrogen starvation and controlled by ATG1, the other is activated by proteasome inhibitors such as MG132 in an ATG1-independent manner (Marshall et al., 2015). When the proteasome is blocked by inhibitors, the proteasome complex is ubiquitylated and recognized by proteaphagy receptors, RPN10 in *Arabidopsis*, to initiate the assembly of autophagosomes (Marshall et al., 2015). RPN10 is a ubiquitin-binding proteasome subunit which can bind the ubiquitylated proteasome through a UIM (Ubiquitin-interacting motif) and ATG8 *via* another UIM-related sequence (Marshall et al., 2019). In the *Arabidopsis rpn10* mutant, inhibitor-induced proteaphagy is blocked (Marshall et al., 2015).

#### Aggrephagy

Nonfunctional proteins are also degraded by selective autophagy in the form of aggregates, termed aggrephagy, with ubiquitin-chains acting as the marker for the degradation (Yoon and Chung, 2019). p62/SEQUESTOSOME 1 (SQSTM1) and Neighbor of BRCA 1 (NBR1) in mammals and Cue5 in yeast are aggrephagy receptors which simultaneously bind ATG8 *via* an AIM and poly-ubiquitin chains *via* a ubiquitin-binding domain (Pankiv et al., 2007; Lu et al., 2014). A homolog of NBR1 has been found in plants. Plant NBR1 contains an N-terminal PB1 (Phox and Bem1p) domain and binds to ubiquitin and ATG8 simultaneously, suggesting conserved mechanisms of aggrephagy in mammals/yeast and plants (Svenning et al., 2011). In *Arabidopsis* undergoing heat stress, mutation of *NBR1* results in the accumulation of ubiquitylated insoluble proteins (Zhou et al., 2013). Also, heat stress can induce the association of NBR1 and ATG8 with accumulated cytoplasmic protein aggregates, suggesting that NBR1 is a plant aggrephagy receptor essential for maintaining proteostasis (Jung et al., 2020).

#### Chlorophagy

The chloroplast is a unique organelle found only in plants and algae, which mainly performs the functions of material and energy metabolism through photosynthesis and signal transduction (Jarvis and López-Juez, 2013). Damaged or redundant chloroplasts are degraded through selective autophagy, called chlorophagy. For example, under darkness or in senescent leaves, chloroplasts become redundant and are transported to the vacuole for recycling *via* an autophagy-dependent process (Wada et al., 2009). Similarly, chloroplasts damaged by strong light or ultraviolet-B are also subjected to degradation through autophagy (Izumi et al., 2017). Chlorophagy happens in many ways, including the whole chloroplast, the Rubisco containing body (RCB), the ATI1-GFP labels plastid-associated body (ATI-PS body), and the small starch granule-like structures pathways (Izumi et al., 2019; Nakamura et al., 2019; Zhuang and Jiang, 2019).

The whole chloroplast pathway includes the ubiquitylation of damaged chloroplasts by the cytosolic ubiquitin ligase PUB4 (Plant U-BOX Protein 4). The ubiquitylated chloroplasts are then encapsulated into ATG8-decorated autophagic vesicles and delivered to the vacuole (Izumi et al., 2017). In contrast, during the early stages of senescence, small double-membrane spherical structures called RCBs, containing stromal proteins such as rubisco but not the chloroplast envelope nor thylakoid proteins, bud from chloroplasts (Ishida et al., 2008). The RCBs may arise from the fission of stroma-filled tubules that protrude from the chloroplast, in which the ESCRT component CHMP1 (Charged Multivesicular Body 1) may play an important role, and are degraded in the vacuole through autophagy (Spitzer et al., 2015; Marshall and Vierstra, 2018). A third pathway of chlorophagy involves plant-specific proteins, ATI1 and ATI2 (ATG8-interacting protein 1 and 2), which localize to the ER- and plastid-derived bodies (Michaeli et al., 2014). These ATI1-decorated plastid bodies contain plastid membrane proteins from the outer envelope or thylakoids and are delivered to the vacuole through the autophagy machinery (Michaeli et al., 2014). Although both come from plastids, RCBs and ATI1-decorated bodies contain different plastid proteins, suggesting two distinct pathways, and function in different developmental stages or environmental conditions. Starch granules exist in the chloroplasts and serve as a carbon reservoir (Malinova et al., 2018). But a small starch granule-like structure (SSGL) is also observed in the cytosol and is sequestered into the autophagic bodies (Wang et al., 2013), indicating that the SSGLs derived from chloroplasts may be degraded by autophagy. Consistent with this idea, silencing of *ATG6* can reduce the number of vacuole-localized SSGLs, suggesting that the degradation of SSGLs is ATG-dependent (Wang et al., 2013).

#### Pexophagy

Plant peroxisomes are ubiquitous organelles housing various metabolic reactions including the glyoxylate cycle, photorespiration, and the β-oxidation of fatty acids (Su et al., 2019). The types of metabolic reactions occurring in peroxisomes change according to the plant developmental stage (Young and Bartel, 2016). For example, peroxisomes are enriched in enzymes of the glyoxylate cycle as they are necessary for seed germination in early developmental stage (Hu et al., 2012). However, in green seedlings, these enzymes are eliminated and replaced by those required for photorespiration (Lingard et al., 2009). It has been demonstrated that this remodeling process requires the involvement of selective autophagy of peroxisomes, termed pexophagy (Bartel et al., 2014).

Peroxisomes are the major site of reactive oxygen species (ROS) production, making them vulnerable to oxidative damage. The peroxisomes also contain multiple antioxidative enzymes to remove excess ROS, such as catalase that specifically degrades H_2_O_2_ (Mhamdi et al., 2012; Su et al., 2018). Peroxisomal catalase detoxifies H_2_O_2_ by converting it to water and molecular oxygen, but catalase itself is also susceptible to damage by H_2_O_2_ (Anand et al., 2009). Aggregates of inactive catalase accumulate in peroxisomes of autophagy-deficient seedlings, and *atg2* mutants show clustered, damaged peroxisomes, indicating that damaged peroxisomes are degraded by pexophagy (Shibata et al., 2013; Yoshimoto et al., 2014). Moreover, other *atg* mutants (*atg5*, *atg7*, *atg10*, and *atg12)* display increased accumulation and aggregation of peroxisomes in guard cells. Additionally, these mutants display increased ROS levels and impaired stomatal opening, indicating that autophagy regulates guard cell ROS homeostasis and stomal opening (Yamauchi et al., 2019).

Pexophagy seems to occur at a higher rate than other types of selective autophagy and is tissue-dependent. For example, pexophagy is active in above ground parts of the plant but not in the roots, possibly because of the higher photorespiratory activity in green tissue (Yoshimoto et al., 2014). However, it has recently been shown that pexophagy in roots is involved in the regulation of glucose-mediated root meristem activity through mediation of auxin biosynthesis (Huang et al., 2019a). In the roots of wild-type plants, high levels of glucose induce ROS accumulation. On the one hand, ROS oxidize active IAA (indole-3-acetic acid). On the other hand, constitutive pexophagy is enhanced by high levels of ROS to attenuate the synthesis of IAA, in turn reducing root meristem activity (Huang et al., 2019a). However, in *atg5* and *atg7*, the autophagy deficiency disrupts transmission of the high-glucose signal to the peroxisomes, enhancing the activity of IAA and the root meristem, resulting in longer primary roots than in wild type under high glucose conditions (Huang et al., 2019a).

How peroxisomes are marked for degradation is not yet clear in plants. In yeast, the AIM-containing pexophagy receptor (Atg30 in *Pichia pastoris* and Atg36 in *Saccharomyces cerevisiae*) binds to Pex3, a peroxisome membrane protein, to recruit the autophagic machinery by interacting with Atg8 and Atg11 (Farré et al., 2013). In *Arabidopsis*, two peroxisomal membrane proteins, PEX6 and PEX10, have been shown to interact with ATG8 *via* an AIM, suggesting that they may participate in the initiation of pexophagy (Xie et al., 2016).

#### Autophagic Degradation of Nuclear Proteins

As the central organelle of the eukaryotic cell, the nucleus plays a key role in maintaining genomic integrity and controlling gene expression. Cells require a strategy to eliminate undesirable nuclear proteins and components when suffering stress or pathogen infection. The mechanisms of selective autophagy of nuclear components including nuclear envelope components, DNA, RNA and nucleoli, termed nucleophagy, are conserved from yeast to mammals (Klionsky et al., 2008; Papandreou and Tavernarakis, 2019). However, for a long time, no evidence demonstrated that nuclear autophagy occurs in plants. Recent research has shown that the accumulation of a geminivirus nuclear protein C1 induces autophagy. ATG8h interacts with C1 and translocates it from the nucleus to the cytoplasm, through the XPO1-mediated nuclear export-dependent pathway (Li et al., 2019a). Mutation of the AIM in C1 abolishes its interaction with ATG8. Degradation of C1 is blocked by autophagy inhibitors and the deletion of *ATG8h*, *ATG5*, or *ATG7* (Li et al., 2019a). This discovery was the first to reveal that autophagy is involved in the degradation of nuclear proteins in plants.

Moreover, the BRI1-EMS Suppressor 1 (BES1), a transcription factor which positively regulates brassinosteroid signaling, is ubiquitylated and interacts with DSK2A to be degraded in a DSK2- and core ATG-dependent manner. DSK2A is an autophagy adaptor which contains a ubiquitin-like domain, two AIMs, and a ubiquitin-associated domain (Nolan et al., 2017). Binding of DSK2 to ATG8 is regulated by BIN2 kinase, which phosphorylates DSK2 near the AIM domains to enhance its binding ability to ATG8 (Nolan et al., 2017). However, it is not clear whether BES1 is degraded before or after it enters the nucleus.

#### Other Selective Autophagy

In addition to the above described selective autophagy pathways, there are also other types of selective autophagy found in plants cells. For example, in *Arabidopsis*, a selective autophagy pathway involved in rRNA turnover which is dependent on ATG5 has been reported (Floyd et al., 2015). In addition, it has been shown recently that mammalian NUFIP1 is a ribophagy receptor required for the selective autophagy of ribosomes during starvation conditions (Wyant et al., 2018). A homolog of mammalian NUFIP1 exists in *Arabidopsis,* but further investigation is needed to show whether *Arabidopsis* NUFIP1 is also involved in ribophagy (Rodor et al., 2011).

Recently, a new class of ATG8 interactors has been described that harbor a UIM-like domain to interact with ATG8 in plants, yeast, and humans (Marshall et al., 2019). Therefore, it is likely that new selective autophagy pathways may be discovered in the near future. These various kinds of selective autophagy pathways ensure that plant cells can specifically remove useless or damaged cell components efficiently to maintain cell viability and enable plant survival during environmental stresses.

## The Function of Autophagy in Plants

### Autophagy During Growth and Development

Autophagy is a highly conserved process which plays significant roles in controlling overall plant development, metabolism, senescence, and stress responses (Janse van Rensburg et al., 2019). Under normal growth conditions, basal levels of autophagy are essential for maintaining cellular homeostasis. Although most *atg* mutants are not fatal and are able to complete their life cycle, they do show clear growth defects. The *Arabidopsis atg4*, *atg5*, *atg7*, *atg10*, and *atg12* mutants all display hypersensitization to nitrogen and carbon starvation and premature senescence compared with wild type (Phillips et al., 2008; Chung et al., 2010). Under nitrogen starvation, *atg5*, *atg7*, and *atg10* seedlings show severely slowed leaf emergence and expansion as well as chlorotic leaves. Similar defects are seen with the *atg4*, *atg5*, *atg7*, and *atg12* mutants under carbon starvation (Phillips et al., 2008; Chung et al., 2010). In contrast, in *Arabidopsis*, overexpression of *ATG5* and *ATG7* stimulates the lipidation of ATG8, autophagosome formation, and autophagic flux, concomitant with increased growth, seed set, and seed oil content (Minina et al., 2018). Overexpression of *ATG8* in *Arabidopsis* not only increases autophagosome number and promotes autophagic activity, but also improves nitrogen remobilization efficiency and grain filling (Chen et al., 2019b). These results indicate that increased autophagy improves plant productivity.

#### The Role of Autophagy in Plant Reproductive Development

In flowering plants, the tapetum provides nutrients and lipids to developing microspores and pollen grains where autophagy is proposed to play an important role. Autophagosome structures and vacuole-enclosed lipid bodies have been observed in post-meiotic tapetum cells during pollen development. These structures disappeared in the autophagy defective mutant *Osatg7*. Moreover, the mutant fails to accumulate lipid and starch in pollen grains and shows complete male sterility, indicating that autophagy is induced in tapetum cells and is essential for another development in rice (Kurusu et al., 2014). Using fluorescent protein-tagged AtATG8 as a marker, it has been shown that the number of autophagosomes increases rapidly at the uninucleate stages in the tapetal cells during anther development (Hanamata et al., 2019). In the anthers of the *Osatg7* mutant, the endogenous levels of active-forms of gibberellins (GAs) and cytokinin, trans-zeatin, are significantly lower than that in wild type, indicating that autophagy may regulate phytohormone metabolism during rice anther development (Kurusu et al., 2017).

During seed development in *Arabidopsis*, almost all *ATG* genes are upregulated in siliques (Di Berardino et al., 2018). *ATG8f* is strongly expressed in the phloem companion cells of pericarps and in the embryo. Moreover, GFP-ATG8 labelled autophagosomes have been observed in seed embryos (Di Berardino et al., 2018). In maize, many *ATG* genes are induced in the endosperm, and the ATG8-PE adducts accumulate in the maize endosperm beginning 18 days after pollination (DAP) with continuing increases until 30 DAP, indicating that autophagy is involved in endosperm development (Chung et al., 2009). The rice *Osatg7-1* mutant also produces smaller seeds with a chalky appearance and lower starch content. During seed maturation of *Osatg7-1*, the starch degradation pathways are activated abnormally in the endosperm, indicating that autophagy plays critical roles in metabolic regulation in the endosperm (Sera et al., 2019).

Additionally, autophagy can regulate the nutrient supply during seed development (Chen et al., 2019a). In *atg5-1*, iron translocation from vegetative organs to seeds is severely decreased during seed formation. It was also shown that the translocations of zinc and manganese to the seeds are also dependent on autophagy (Pottier et al., 2019). In *Arabidopsis atg* mutants, the amount of 12S globulins in seeds is decreased, but the amount of 12S globulin precursors are increased, suggesting that autophagy is involved in delivering the precursors to the protein storage vacuoles (PSVs), where the precursors are processed into their mature forms (Di Berardino et al., 2018).

#### Roles of Autophagy in the Regulation of Lipid Metabolism

Lipids in membranous organelles are important substrates for energy production and as cellular structural materials. Before being used directly for β-oxidation, fatty acids are first stored in lipid droplets (LDs) in the form of triacylglycerol (TAG) (Fan et al., 2014; Fan et al., 2017). The LDs are hydrolyzed by lipolysis to supply the cell with fatty acids. In mammalian cells, the degradation of LDs is carried out through a selective autophagy pathway, termed lipophagy (Wang, 2016; Zechner et al., 2017). In comparison with yeast and mammals, the mechanisms of lipophagy have been less well characterized in plants (van Zutphen et al., 2014; Jaishy and Abel, 2016).

During pollen maturation, LDs containing TAGs in the tapetum are necessary as a supply of lipid components (Kurusu et al., 2014). In tapetum cells of rice, LDs enclosed in vacuoles have been detected, and LD-like structures were more abundant in the cytoplasm of *Osatg7* and *Osatg9* mutants than that of wild type, indicating that LDs in plant may also be degraded through lipophagy (Kurusu et al., 2014). Furthermore, lipidomic analysis has shown impairment of phosphatidylcholine (PC) editing and lipid desaturation in the anthers of these mutants during pollen maturation, indicating the involvement of autophagy in the regulation of lipid metabolism during plant development (Hanamata et al., 2014; Kurusu et al., 2014). In *Arabidopsis*, the autophagy of cellular organelles can provide a source of fatty acids for TAG synthesis under normal and starvation conditions, indicating that autophagy can promote TAG synthesis. Under normal conditions, TAGs stored in LDs are hydrolyzed by SDP1 (sugar-dependent 1), a lipase responsible for the initiation of TAG catabolism (Eastmond, 2006). But under nutrient starvation, lipophagy is induced to degrade the LDs for energy production (Fan et al., 2019). In the *atg5* mutant, peroxisomal and ER proteins involved in very long chain fatty acid synthesis and β-oxidation are up-regulated, and the concentrations of sphingolipids, phospholipids and galactolipids are changed, indicating that lipid metabolism is severely affected in autophagy mutants and that autophagy can play a role in the control of plant lipid metabolism in addition to regulating TAG synthesis and LD degradation (Havé et al., 2019).

#### The Roles of Autophagy in Plant Root Development

The regulation of primary and secondary root development is important for the establishment of root systems. It has been recently demonstrated that autophagy is involved in the regulation of plant root development. Under phosphate starvation, phosphorylation and activation of PUB9 (U Box/Armadillo Repeat-Containing E3 ligase) by ARK2 (S-Domain receptor Kinase) is responsible for ubiquitination of either AUX/IAA (auxin/indole-3-acetic acid) proteins or other repressors of auxin accumulation. These auxin repressors are subsequently selectively targeted to the autophagosome for degradation, releasing ARFs (auxin response factors) or other signals to promote auxin accumulation and lateral root development. The inhibition of autophagy by 3-MA leads to disruption of both lateral root growth and auxin accumulation in the roots under phosphate starvation, suggesting autophagy plays an important role in root growth (Deb et al., 2014; Sankaranarayanan and Samuel, 2015). In untreated *Arabidopsis*, overexpression of *AtATG8f* results in plants with fewer lateral roots than control plants, despite similar primary root lengths. The application of zeatin results in reduced length of primary roots and reduced number of lateral roots in both plants. However, the extent of inhibition was remarkable in length of the primary roots but similar in number of the lateral roots in overexpression of *AtATG8f* plants. Together these data indicate that ATG8 affects the cytokinin-mediated regulation of root architecture (Slavikova et al., 2008).

Glucose is a key nutrient signal regulating root meristem activity in plants. It has recently been found that autophagy is involved in glucose-regulated root meristem maintenance (Pacifici et al., 2015; Huang et al., 2019a). Under high glucose conditions, accumulation of ROS oxidizes active IAA and impairs root meristem activity and growth. However, in *atg* mutants, the transmission of the high-glucose signal to the peroxisomes is disrupted, alleviating ROS-oxidized IAA, causing increased root growth (Huang et al., 2019a). These data demonstrate that constitutive autophagy regulates the production of ROS and IAA by the peroxisome to modulate root meristem activity. Autophagy was also found to play a key role in the hydrotropic curvature of *A. thaliana* roots. Autophagosomes accumulate in the root curvature of wild type seedlings after being transferred to a water potential gradient stress system called Normal Medium-Water Stress Medium (NM-WSM). In contrast, *atg2*, *atg5*, *atg8b*, *atg8i*, and *atg9* mutants do not show hydrotropic curvature in the NM-WSM system. During the hydrotropic response, H_2_O_2_ also accumulates in the root curvature at a similar rate as the autophagosomes (Jiménez-Nopala et al., 2018), suggesting that oxidative stress, and specifically H_2_O_2,_ induced during the hydrotropic response in NM-WSM regulates autophagy.

In *Populus trichocarpa*, *ATG* genes and the ATG8 protein are expressed at different stages during root or stem primary and secondary development. Ultrastructural observations have revealed that autophagosomes accumulate during differentiation of xylem in roots (Wojciechowska et al., 2019). These results exhibit autophagy is involved in root morphogenesis.

#### The Involvement of Autophagy During Senescence

The involvement of autophagy during senescence has been supported by the observation that many *ATG* genes are upregulated in older leaves. In *Arabidopsis*, 15 *ATG* genes are upregulated during senescence (Breeze et al., 2011). In maize, ATG8 lipidation, which is a marker for higher levels of autophagy activity, is highly accumulated in the yellowing area of senescent leaves (Chung et al., 2009). ATG11, which links the ATG1/ATG13 complex to autophagic membranes, also plays a critical role in senescence-induced mitophagy in *Arabidopsis*. The senescence-induced breakdown of mitochondria-resident proteins and mitochondrial vesicles occurs *via* an autophagic process requiring ATG11 and other ATG components (Li et al., 2014). In *atg* mutants, defective autophagy results in early senescence and PCD irrespective of nutrient conditions. The salicylic acid (SA) signaling pathways are required for this senescence/PCD phenotype (Yoshimoto et al., 2009). Application of a SA agonist cannot induce the senescence/PCD phenotype in *atg npr1* mutants, indicating that induction of this phenotype in *atg* mutants requires the SA signal transducer NPR1 (nonexpressor of pathogenesis-related genes 1). Autophagy is induced by a SA agonist *via* NPR1, thus indicating that autophagy can operate as a negative feedback regulator of SA signaling to limit senescence and PCD (Yoshimoto et al., 2009). In conclusion, autophagy can be induced at different developmental stages to perform different physiological functions in plants.

### Autophagy in the Plant’s Response to Abiotic Stress

As immovable organisms, plants need to cope with various environmental changes, including nutrient starvation, salt, drought, and heat stresses (Han et al., 2014; Shen et al., 2014; Qi et al., 2018; Hilker and Schmülling, 2019). To respond appropriately to these stresses, plants have evolved various distinct signaling and regulatory mechanisms (Hilker and Schmülling, 2019). It has been shown that autophagy is involved in removing damaged proteins and cellular components produced in response to environmental stresses (Wang et al., 2018b). Furthermore, Autophagy is induced by various abiotic stresses and autophagy-defective mutants are hypersensitive to these stress conditions (Wang et al., 2018b). For example, macroautophagy-defective *RNAi*-*AtATG18a* plants are more sensitive than wild type plants to methyl viologen, a ROS inducer, and accumulate more oxidized proteins (Xiong et al., 2007). In this section, we review the roles of autophagy in plant stress response and the underlying mechanisms.

#### Autophagy can be Induced by Abiotic Stress

In response to starvation, autophagy is upregulated to facilitate the recycling of cellular material and remobilization of nutrients. The evidence that autophagy is involved in nitrogen remobilization from leaves into seeds comes from research using ^15^NO3^−^. It was shown that ^15^N remobilization is decreased significantly in *ATG18a* RNAi, *atg5*, and *atg9* mutants compared with wild type plants under nitrogen starvation (Guiboileau et al., 2012). Moreover, more ammonium, amino acids, and proteins accumulate in the rosette leaves of these *atg* mutants than in wild type (Guiboileau et al., 2013; Chen et al., 2019b). In maize, the growth rate and seed yield of the *atg12* mutant is decreased significantly in low nitrogen conditions but not in sufficient nitrogen conditions. However, even under nitrogen rich conditions, the seed yield and ^15^N reallocation into the seeds are much lower in *atg12* than in wild type (Li et al., 2015a). Recently, a study illustrated that the overexpression of *ATG18a* in apple results in increased tolerance to nitrogen starvation due to increased autophagy under these conditions (Sun et al., 2018). Additionally, under carbon starvation, the *Arabidopsis atg5* and *atg7* mutants exhibit delayed growth, reduced amino acid levels, increased respiration, and decreased flux to net protein synthesis. These results highlight the importance of autophagy in cellular metabolism and energy homeostasis in *Arabidopsis* seedlings (Avin-Wittenberg et al., 2015).

Heat stress is one of the most dangerous threats for plant growth, causing the misfolding and denaturing of normal proteins, which can be degraded through autophagy (Zhang et al., 2010; Fu et al., 2015). It has been recently shown that the NBR1-mediated selective autophagy pathway is involved in the degradation of denatured or otherwise damaged proteins caused by heat stress (Zhou et al., 2013). The *nbr1* mutant shows reduced heat tolerance and increased accumulation of insoluble, detergent-resistant and highly ubiquitinated proteins under heat stress, implying that the autophagy adaptor NBR1 targets the ubiquitinated protein aggregates for degradation under stress conditions (Zhou et al., 2013). As further evidence that autophagy is involved in the heat stress response, the expression of *ATG* genes and the accumulation of autophagosomes are induced by heat stress (Wang et al., 2015). Moreover, it was shown that ATG8 coimmunoprecipitates with different classes of heat shock proteins (HSP90s, HSP101, and small HSP17.6) in plants. Defective autophagy causes accumulation of these HSPs, suggesting that autophagy promotes the degradation of HSPs and unfolded proteins (Sedaghatmehr et al., 2019). Additionally, in *Arabidopsis*, high-temperature stress (30°C) promotes autophagy in both anther wall cells and microspores in developing anthers of plants, while *atg* mutants (*atg2-1*, *atg5-1*, *atg7-2*, and *atg10-1*) have visibly impaired pollen development and anther dehiscence, suggesting that autophagy functions in tapetum degeneration and pollen development during high-temperature stress (Dündar et al., 2019).

Drought and salt are two common environmental stresses that plants may encounter (Golldack et al., 2014; Zheng et al., 2017; Zhang et al., 2018). The involvement of autophagy in response to drought stress was first elucidated in *Arabidopsis*, indicated by the upregulation of *ATG18a* during drought conditions (Liu et al., 2009). In *Arabidopsis*, the *atg5*, *atg7*, and *ATG18a* RNAi mutants display hypersensitization to drought stress (Zhou et al., 2013). On the other hand, overexpression of *ATG18a* in apple results in higher autophagy activity and increased drought tolerance, indicating the key role for autophagy in responses to drought (Sun et al., 2018). Recently, *Medicago truncatula* dehydrin MtCAS31 (cold acclimation-specific 31) was found to interact with both ATG8a and a negative drought stress regulator MtPIP2;7 to improve drought tolerance by facilitating autophagic degradation of MtPIP2;7, demonstrating that MtCAS31 functions as a positive regulator of drought-induced autophagy (Li et al., 2019b). Consistent with this idea, the *Arabidopsis atg2* and *atg7* mutants contain more oxidized proteins and are hypersensitive to both salt and osmotic stresses (Luo et al., 2017). Under salt stress, autophagy is required for Na^+^ sequestration in the central vacuole in root cortex cells (Luo et al., 2017). Interestingly, autophagy induction by nutrient starvation and salt stress, but not osmotic stress, can be blocked by NADPH oxidase inhibitors (Liu et al., 2009). These results suggest that autophagy is also involved in the oxidative stress response and can be induced by NADPH oxidase-dependent or -independent pathways.

#### The Regulation of Autophagy in Response to Abiotic Stresses

**Figure 2:**
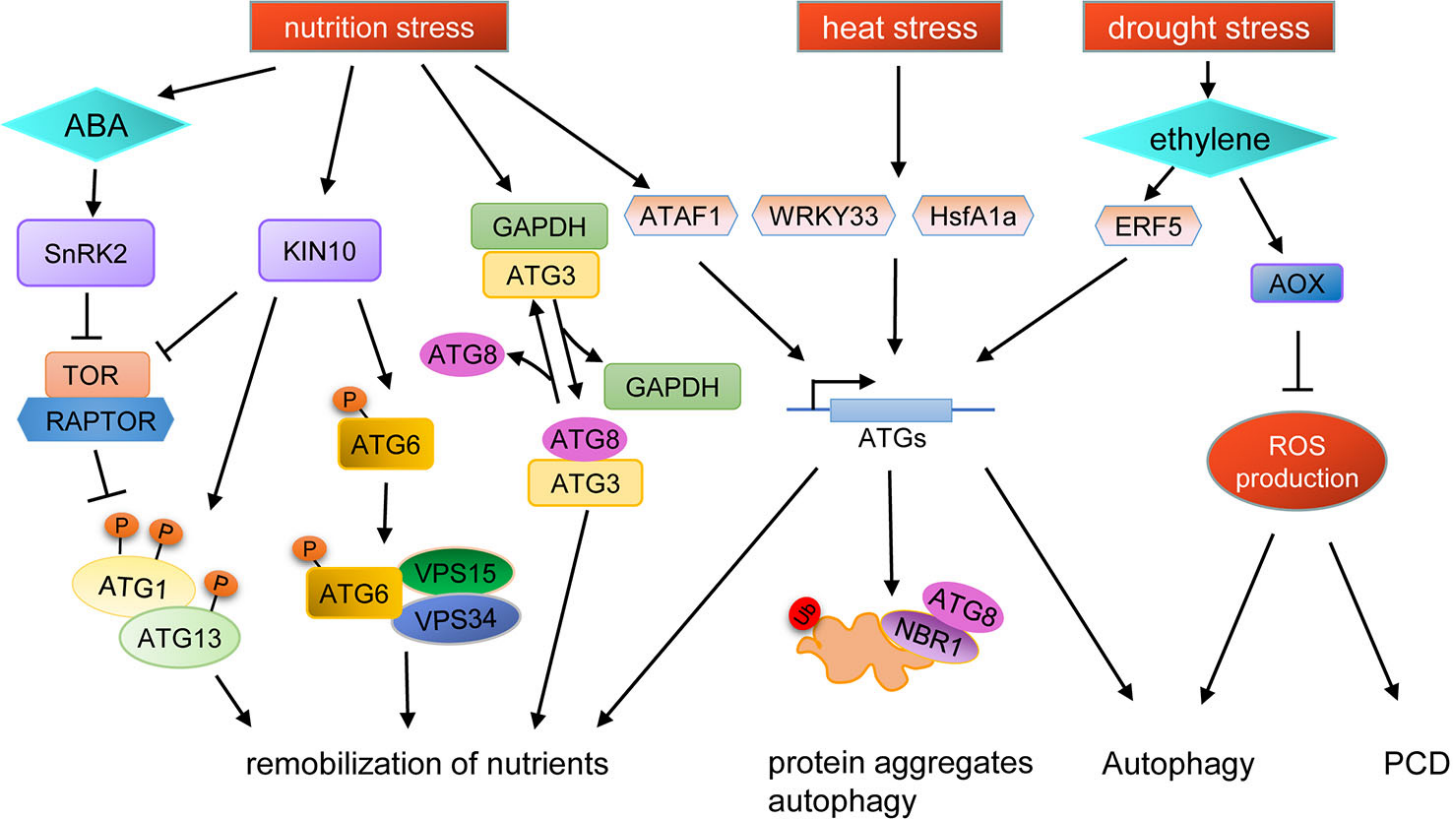
Schematic diagram of autophagy regulation under various stresses. KIN10, a catalytic subunit of SnRK1, is activated by stresses to phosphorylate TOR-interacting proteins, in turn inhibiting TOR kinase activity and activating ATG1. Additionally, active KIN10 can phosphorylate ATG6 and activate the PI3K complex to induce autophagy by an ATG1 kinase independent pathway. Under nutrient starvation stress, SnRK2 is activated by ABA to inhibit the activity of the TOR complex, and the expression of *ATG* genes are induced by the transcription factor ATAF1, resulting in the promotion of autophagy. Nutrient starvation stress can also cause the dissociation of GAPDH and ATG3, thus promoting the binding of ATG8 and ATG3. The expression of *ATG* genes are induced by WRKY33 and HsfA1a under heat stress to promote the degradation of protein aggregates through autophagy. Autophagy can also be induced by drought stress in an ethylene-dependent manner. Moreover, autophagy is also regulated by ROS levels during drought stress, in which AOX plays an important role.

TOR kinase is a major switch in controlling autophagy, and the phenotypes of TOR knockout and knockdown mutant lines indicate that TOR plays important roles in nutrient stress responses in plants (Menand et al., 2002; Liu and Bassham, 2010). Recently, it was shown that ATG13 in plants can be phosphorylated directly by TOR (Van Leene et al., 2019), suggesting that autophagy may be involved in stress response in plants through similar regulatory mechanisms as in yeast and mammals. TOR activity is also regulated by SnRK1, which is repressed by sugars and activated under energy-deficient conditions, such as darkness or under biotic and abiotic stresses (Baena-González et al., 2007). Thus, during responses to environmental stresses, TOR activity is regulated by SnRKs, subsequently, regulating the autophagy process. Interestingly, under prolonged fixed-carbon starvation, the active KIN10 subunit of SnRK1 phosphorylates ATG6 to activate the PI3K complex, which bypasses the requirement of the ATG1 kinase complex for autophagy initiation (Huang et al., 2019a; Huang et al., 2019b) (**Figure 2**).

Another regulatory pathway of autophagy in plants is mediated by glyceraldehyde-3-phosphate dehydrogenase (GAPDH) (Masclaux-Daubresse et al., 2017). In tobacco, cytosolic GAPDHs (GAPCs) interact with ATG3, resulting in the inhibition of the ATG3-ATG8 interaction, thereby preventing ATG3-dependent ATG8-PE conjugation (Han et al., 2015). In *Arabidopsis*, the *gapc1* mutant shows enhanced autophagy compared to wild type (Henry et al., 2015).

Autophagy is also controlled at the transcriptional level (Wang et al., 2019a). In yeast and mammals, Rph1/KDM4, a histone demethylase protein, is a repressor of the transcription of several *ATG* genes and thus of autophagy induction. Under nitrogen starvation conditions, Rph1/KDM4 can be phosphorylated by Rim15 to relieve the inhibition of *ATG* gene expression (Bernard et al., 2015). In *Arabidopsis*, under carbon starvation, a NAC transcription factor ATAF1 (*Arabidopsis* transcription activation factor 1) induces *ATG* gene expression to promote the carbon starvation response (Garapati et al., 2015). Under heat stress, downregulation of *WRKY33* expression in tomato plants causes an increase in sensitivity to heat stress and reduces the expression of several *ATG* genes, suggesting that WRKY33 participates in the heat stress response through transcriptional regulation of autophagy (Zhou et al., 2014). Moreover, in tomato, the expression of *ATG10* and *ATG18f* are regulated by HsfA1a (heat shock transcription factor A1a) to activate autophagy under drought stress (Wang et al., 2015).

#### Autophagy and Abiotic Stress are Connected by ROS

Many kinds of abiotic stresses can induce ROS production, which can act as a signal molecule for stress response. ROS is produced in many compartments of plant cells, such as the plasma membrane, mitochondria, chloroplasts, and peroxisomes (Mhamdi et al., 2012). Recent studies have shown that ROS acts as a link between autophagy and abiotic stress (**Figure 2**). For example, plasma membrane-associated NADPH oxidase, an important source of ROS, is necessary for plant tolerance to submergence and autophagy activation (Chen et al., 2015). In *Solanum lycopersicum*, mitochondrial alternative oxidase (AOX) may regulate autophagy by controlling ROS production during drought stress (Zhu et al., 2018). ROS production can be limited by AOX, and overexpression of AOX results in increased drought tolerance in tomato, concomitant with increased autophagy activity during drought (Selinski et al., 2018; Zhu et al., 2018). In *AOX1a-RNAi* plants, ethylene-induced drought tolerance is disrupted and the level of autophagy is decreased, accompanied by higher levels of H_2_O_2_, which results in rapid programmed cell death (PCD). Pharmacological scavenging of H_2_O_2_ in the *aox19* mutant induces autophagosome accumulation under drought stress, indicating that AOX-dependent ROS signaling is critical in triggering autophagy (Zhu et al., 2018). In summary, AOX can reduce the ROS burst caused by drought and subsequently, the low ROS levels promote autophagy to aid drought tolerance in tomato (Zhu et al., 2018).

ROS can modulate autophagy by causing damage to cellular components (Signorelli et al., 2019). Upon treating *Arabidopsis* roots with exogenous H_2_O_2,_ autophagy is induced to degrade oxidized proteins (Xiong et al., 2007). In *Chlamydomonas*, a deficiency in carotenoid synthesis triggers autophagy in the light, but not in the dark, indicating that ROS produced in the chloroplastic electron chain are able to induce autophagy (Pérez-Pérez et al., 2012). Redox status can also modulate autophagy by regulating ATG4 activity. In *Arabidopsis*, cleavage of ATG8f by ATG4 is dramatically decreased with increasing concentrations of H_2_O_2_, but ATG4 activity can be restored with the addition of the reducing agent DTT (dithiothreitol) (Woo et al., 2014). Similarly, in *Chlamydomonas reinhardtii*, DTT treatment promotes monomeric and active ATG4, whereas NF (norflurazon) treatment results in oxidation and aggregation of ATG4, rendering it inactive. In the *C. reinhardtii* carotenoid-less mutant *lts1-204* that lacks phytoene synthase, a shift from dark to light conditions leads to increased ROS levels and induction of autophagy. Moreover, in the *lts1-204* mutant, the abundance of oligomeric ATG4 is significantly increased, concomitant with accumulation of lipidated ATG8. Together, these data indicate that redox status regulates autophagy through the oxidation and inactivation of ATG4 under stress conditions (Pérez-Pérez et al., 2016).

Autophagy in turn contributes to the antioxidant system through degradation of oxidative damaged organelles, such as mitochondria, chloroplasts, and peroxisomes (Signorelli et al., 2019). Consistent with the role of autophagy in oxidative stress, *atg2* and *atg5* mutants exhibit enhanced accumulation of H_2_O_2_ (Yoshimoto et al., 2009). Additionally, silencing of *ATG18a* in *Arabidopsis* results in increased oxidative damage and subsequently, hypersensitivity to oxidative stress (Yoshimoto et al., 2009; Minibayeva et al., 2012). In autophagy-deficient seedlings, inactive catalases accumulate in clustered peroxisomes, implying that pexophagy actively clears damaged peroxisomes (Shibata et al., 2013). ROS are key signaling molecules for promoting stomatal closure in response to diverse environmental stresses. The disruption of other ATGs, such as ATG2, ATG5, ATG7, ATG10 or ATG12, promotes the aggregation of peroxisomes in guard cells. These mutants exhibit reduced activity of the ROS scavenger catalase, resulting in impairment of stomatal opening in response to light and low CO_2_ concentrations (Yamauchi et al., 2019). In summary, autophagy and ROS production are two important cellular physiological processes that work cooperatively during the plant’s response to abiotic stresses, enabling plants to cope with various environmental challenges.

#### Crosstalk Between Autophagy and Phytohormones

Phytohormones play important roles in the regulation of plant development and environmental stress response. The *atg* mutants defective in autophagy have been used to uncover the connection between the phytohormone pathway and autophagy (Signorelli et al., 2019) (**Figure 2**). *Arabidopsis* TSPO (Tryptophan-rich Sensory Protein) can respond to osmotic and salt stresses and is induced by abscisic acid (ABA) to directly bind to ATG8 in an AIM-dependent manner. In wild-type, ABA-induced TSPO is degraded quickly, while in the *atg5* mutant, degradation of ABA-induced TSPO is inhibited. Moreover, ABA-induced TSPO is stabilized in the presence of autophagic inhibitors, such as 3-MA, and the vacuolar Cys protease inhibitor E64d. Thus, these data indicate that autophagy is involved in the ABA-mediated stress response (Guillaumot et al., 2009; Vanhee et al., 2011). In addition, ATG8-interacting protein 1 and 2 (ATI1 and ATI2) are involved in ABA-mediated germination (Avin-Wittenberg et al., 2012; Honig et al., 2012). Under unstressed conditions, TOR kinase phosphorylates PYL ABA receptors, disrupting PYL association with ABA and PP2C phosphatase effectors, leading to inactivation of SnRK2 kinases (Wang et al., 2018b). Under stress conditions, SnRK2 is activated by ABA to phosphorylate RAPTOR, a component of the TOR complex, resulting in the dissociation and inhibition of the TOR complex (Wang et al., 2018b; Salem et al., 2018). Therefore, as an endogenous messenger during abiotic stresses, ABA is capable of inducing autophagy in multiple ways.

In addition to ABA, the abundance of Brassinazole-resistant 1 (BZR1) is controlled by TOR signaling to maintain growth under carbon starvation conditions in *Arabidopsis*, suggesting crosstalk between autophagy and BR signaling (Zhang et al., 2016). *ATG* gene expression and autophagosome formation are decreased in the *bzr1* mutant but are enhanced in *BZR1* overexpressing plants after BR treatment. In the *atg2* and *atg6* mutants, initiation of BR-induced autophagy is disrupted. Additionally, silencing of *BZR1* decreases resistance to nitrogen starvation in tomato (Wang et al., 2019b). As previously described above, BES1 is degraded by a selective autophagy pathway mediated by DSK2 under drought and starvation stresses (Nolan et al., 2017), indicating that BR signaling is also involved in autophagy regulation during stress response.

Ethylene is also a stress response hormone. In *atg5* and *atg9* mutants, a subset of ethylene signaling genes are induced, such as *ETR2* (ethylene response 2) and *CTR1* (constitutive triple response 1), suggesting that ethylene is overproduced in the *atg* mutants (Masclaux-Daubresse et al., 2014). Moreover, under drought stress, ERF5 (ethylene response factor 5) binds to the tomato promoters of *ATG8d* and *ATG18h* to induce autophagy (Zhu et al., 2018). These results suggest that ethylene signaling may also be involved in the regulation of autophagy induced by stresses, although the underlying mechanism requires further investigation.

## Future Perspectives

Autophagy plays an important role in recycling cytosolic material and maintaining cellular homeostasis during growth, development and response to diverse stresses. In recent years, considerable efforts have focused on revealing the mechanisms of autophagy, with great achievements being made in recent decades. Many of the protein components and molecular mechanisms involved in autophagy have been identified and key regulatory factors have also been discovered, such as the TOR complex (Liu and Bassham, 2010) and SnRK1 (Soto-Burgos and Bassham, 2017). Studies on the roles of autophagy in plant stress tolerance have enabled us to understand the positive significance of autophagy on plants.

However, most studies have only described the phenomenon that autophagy is involved in plant development and stress response, leading to several important unanswered questions about the underlying molecular mechanisms that remain to be further investigated. For example, how do cells choose the right autophagy pathways at the proper time when adjusting their life state and how are these autophagy pathways related to each other? During plant development, different tissues undergo different physiological processes, and selective autophagy must cooperate with other regulatory pathways to promote normal development. Therefore, the appropriate switch of autophagy and its coordination with other pathways are crucial processes that require further investigation. Additionally, many of the receptors for selective autophagy are unknown. Thus, it is of great importance to identify and characterize the adaptors/receptors or other new components that mediate the various selective autophagy pathways. Lastly, how the autophagy machinery is activated in response to biotic and abiotic stresses of plants, and how it works cooperatively with other regulatory pathways in response to stress still remain to be elucidated. The study of these scientific issues will enable us to better understand the important physiological process of autophagy.

## Author Contributions

TS, XL, PW and CM designed the concept and wrote the manuscript. MY, YZ and QS contributed to revision of the manuscript. All authors read and approved the submitted version.

## Funding

This study was funded by the National Natural Science Foundation of China (31670073, 31600204, 31770290 and 31970301), the Key Technology Research and Development Program of Shandong (2018GSF121037 and 2018GNC113010), and the China Postdoctoral Science Foundation (2017M612333).

## Conflict of Interest

The authors declare that the research was conducted in the absence of any commercial or financial relationships that could be construed as a potential conflict of interest.
